# Combined Immunodeficiency Associated with Two Novel CARMIL2 Mutations: A Case Series

**DOI:** 10.1007/s10875-025-01956-1

**Published:** 2025-11-10

**Authors:** Saja I. Abu Ghannam, Celina R. Andonie, Tala Mahmoud Hamadna, Lila H. Abu-Hilal, Rabee S. Adwan

**Affiliations:** 1 Medical Graduate at Al-Quds University, Jerusalem, Palestine; 2 Internal Medicine Resident at Al- Makassed Hospital, Jerusalem, Palestine; 3 Faculty of Medicine, Al-Quds University, Pediatrician and Infectious Disease specialist at Al- Makassed Hospital, Jerusalem, Palestine

**Keywords:** Carmil2 mutation, Combined immunodeficiency, Primary immunodeficiency disorders, Visceral leishmaniasis, Novel mutation.

## Abstract

Combined immunodeficiency due to CARMIL2 mutations is a rare autosomal recessive primary immunodeficiency characterized by impaired T-cell activation and function, leading to diverse clinical manifestations. Fewer than 50 cases have been reported worldwide. We describe the clinical and genetic features of five patients from Palestine with homozygous CARMIL2 mutations, including the first documented case of recurrent visceral leishmaniasis associated with this gene defect. This retrospective case series was conducted using whole-exome sequencing to confirm the diagnosis. All patients exhibited significant immunologic symptoms, including chronic dermatitis, cutaneous warts, recurrent respiratory infections, and mucocutaneous candidiasis. Two developed cytomegalovirus-related disease. Genetic analysis revealed two novel homozygous variants: NM_001317026.3:c.1865 C > T (p.Ala622Val) in four patients, and c.1973 C > T (p.Ala658Val) in one. Notably, one adult male developed recurrent visceral leishmaniasis, an unusual presentation not previously reported in the context of CARMIL2 deficiency. Consanguinity was identified in two families. All patients required immunomodulatory therapy, and four were evaluated for hematopoietic stem cell transplantation. This case series underscores the clinical heterogeneity of CARMIL2-associated immunodeficiency and highlights the importance of genetic testing in patients with recurrent or atypical infections, particularly in populations with a high prevalence of consanguinity. The novel link to visceral leishmaniasis expands the known phenotypic spectrum of this condition.

**Supplementary Information:**

The online version contains supplementary material available at 10.1007/s10875-025-01956-1.

## Introduction

Combined immunodeficiencies (CID) is a term used to describe a varied group of primary immunodeficiency disorders (PID) that display deficiencies in either the development or function of T-cells. The malfunction of CD4 + helper T-cells in CID not only affects B-cell activity but also impacts both the cellular and humoral arms of the lymphocyte response system. This dual impairment contributes to the designation of the disorder as “combined”. CIDs emerge from inherent abnormalities in the pathway of lymphocyte differentiation. Severe Combined Immunodeficiency Disease (SCID) is a distinct subtype of CID characterized by the absence of identifiable autologous T-cells in blood and lymphoid tissues. It can result from various mechanisms such as apoptosis of hematopoietic progenitor cells, impaired cytokine or T-cell receptor (TCR) signaling, and other pathways [1, 2]. The prevalence of mutations in genes associated with CID and SCID is notably higher in regions where consanguinity is more widespread, owing to the autosomal recessive nature of these genes [3, 4]. 

Within the field of primary immunodeficiencies, a unique subset has recently come to the forefront- combined immunodeficiency due to CARMIL2 (RLTPR) mutation. CARMIL2, also known as capping protein regulator and myosin 1 linker 2, is a gene that encodes a protein involved in crucial cellular processes, including actin cytoskeleton dynamics, immune response regulation, and cell motility [1]. Mainly, the expression of the CARMIL2 protein is observed in the skin, lymphoid tissue, and gastrointestinal system [2]. The CARMIL2 mutation is a rare autosomal recessive disease that refers to alterations in the genetic sequence of the CARMIL2 gene, resulting in dysfunction in T-cell activation, proliferation, differentiation, function, and memory responses [5, 6]. This genetic abnormality has been implicated in a growing number of clinical conditions, ranging from immunodeficiencies and autoinflammatory disorders to developmental abnormalities and cancer predisposition. Presently, fewer than 50 cases of reported CARMIL2 mutations have been documented globally [1, 6, 7]. The majority of reported symptoms associated with the CARMIL2 mutation involves recurrent respiratory system infections, cutaneous warts, psoriatic rash, recurrent condyloma, molluscum contagiosum, solar urticaria, and different forms of dermatitis. Additional clinical manifestations include failure to thrive, dysphagia, lymphocytic esophagitis, and early-onset inflammatory bowel disease. Moreover, Epstein-Barr Virus (EBV)-related smooth muscle tumors may occasionally occur in patients with CARMIL2 mutations [5, 8]. 

In this paper, we report a series of five cases that were diagnosed with CARMIL2 mutation by using whole exome sequencing (WES). These patients presented with multiple clinical manifestations including recurrent infections, dermatitis, cutaneous warts and abscesses, and esophageal candidiasis. Uniquely, our study sheds light on the first documented case of frequent relapsing visceral leishmaniasis attributed to a CARMIL2 mutation. Notably, it marks the first study in the region, contributing valuable insights into the landscape of healthcare and genetic disorders in Palestine.

## Case Presentations

The first patient was a 37-year-old married male, born in Palestine, who was a known case of recurrent visceral leishmaniasis since 2004. The patient was first diagnosed based on his clinical picture as he presented with documented spiking high-grade fever occurring daily, mainly at bedtime (38 °C), heavy sweating, loss of appetite, and generalized weakness without skin involvement. On physical examination, he had hepatosplenomegaly; the liver was palpable 2 cm below the costal margin, with a span of 14 cm, and the spleen was palpable 3 cm below the costal margin. He also had sublingual and submandibular lymphadenopathy. Upon laboratory testing, he had complete blood cell count findings of pancytopenia. Later on, the patient’s bone marrow biopsy showed macrophages with amastigotes/Donovan bodies which are considered positive pathological evidence for visceral leishmaniasis (Figure S1).

He was treated accordingly with sodium stibogluconate (pentostam) for one month with complete resolution of his symptoms after a few weeks of treatment. The patient had another three relapses with similar clinical presentations, bone marrow biopsy findings, and treatment in 2015, 2017, and 2018 with a complete remission between relapses. Because of the recurrent relapses of his visceral leishmaniasis, immunodeficiency diseases were considered possible diagnoses. Thus, SCID and other severe immunodeficiencies were considered. Immunophenotyping of the patient is demonstrated in the table below (Table S2). Based on these results, SCID or severe immunodeficiency was possible. WES showed CARMIL2 gene mutation NM_001317026.3, c.1865 C > T; p.Ala622Val, which is a type of PID. After confirming his diagnosis, the patient had another three relapses; the last one was in 2020. At that time, he had a severe relapse of his usual visceral leishmaniasis. In addition, he had right-sided vision loss due to Cytomegalovirus (CMV) retinitis, herpes stomatitis, and suspected CMV colitis. Thus, he needed urgent treatment for his acute relapse, with liposomal amphotericin B 5–7 mg/kg, miltefosine, which was hardly made available but unfortunately was not tolerated due to severe GI upset, IVIG 20 g every month regularly, and IV ganciclovir 225 mg BID for 6 weeks. Blood CMV Polymerase chain reaction (PCR) and colonoscopy with biopsy were recommended but were not done for unknown reasons. To address his underlying immunodeficiency, a bone marrow transplant was deemed essential. Tragically, he passed away a few months later before the procedure could be undertaken.

The second and third patients are two siblings born to consanguineous parents. The elder sibling was a 15-year-old female. She presented with many warts on her hands. She also had a history of recurrent pulmonary infections and chronic dermatitis. On physical examination, the patient looked cachectic with multiple well-demarcated, irregular, flesh-colored, variable-in-size warts involving her hands and fingers (Figure S2).

Having a confirmed family history of SCID and CARMIL2 mutation on the maternal side, the patient underwent laboratory testing and a genetic workup. Immunophenotyping results are demonstrated in (Table S2). In light of these results, the likelihood of typical SCID was low; however, the potential for an atypical form or an alternative PID was still under consideration. WES revealed a result of positive CARMIL2 mutation, NM_001317026.3, c.1865 C > T; p.Ala622Val genomic position (Hg38): Chr. 16, 67,683,762. She was maintained on trimethoprim-sulfamethoxazole, itraconazole, and fluorouracil with a salicylic acid solution. However, she was still complaining of multiple warts and recurrent pulmonary infections, so she needed a regular monthly dose of IVIG 20 g as prophylaxis while waiting for a bone marrow transplant.

The younger sibling was a 13-year-old male who had a history of recurrent infections, chronic dermatitis, and recurrent warts, presented with undocumented fever and a productive cough of 4 days duration. He also faced difficulty and pain with swallowing. His immunizations were up-to-date. Upon seeking medical care in an outpatient clinic, his White blood cell count (WBC) was found to be elevated as well as the C-reactive protein (CRP) level. The patient was treated with IV ceftriaxone, azithromycin, and nebulizers; however, the symptoms persisted. Two days later, his WBCs and CRP levels were still high. Thus, the patient was referred to a nearby hospital for further evaluation, where CBC was done again and showed elevated lymphocytes and neutrophils, increased platelet count, and low hemoglobin level. As a workup for leukocytosis, his blood film was performed and showed immature myeloid maturation and atypical lymphocytes. The patient was admitted to a more advanced hospital for further evaluation, where blood and urine cultures were taken, and he was started on intravenous fluids and prophylactic antibiotics. Obtained cultures were negative. A blood film was done and showed microcytic hypochromic red blood cells (RBCs), and atypical lymphocytosis with left-shifted neutrophils, no blasts were seen. Flow-cytometry was ordered to rule out leukemia and immunodeficiency- keeping in mind the positive family history. Immunoglobulin levels were done and demonstrated in (Table S2). In the following few days, abdominal ultrasound was done, with no significant abnormalities, however, it is worth mentioning that the liver size was at the upper limit of normal. Moreover, the patient has undergone a whole-body CT scan, which showed signs of sinusitis and pulmonary infection. On the next day, upper endoscopy was also done, and multiple biopsies were taken (esophageal, gastric, and duodenal). Esophageal biopsy was positive for Candida esophagitis. Gastric biopsy was positive for Helicobacter pylori with associated mild active gastritis as well as moderate chronic gastritis. The patient was tested for CMV and EBV and the results are demonstrated in (Table S2). As the patient has a family history of CARMIL2 mutation and to rule out immunodeficiency, WES was ordered as well. The patient was found to have a homozygous mutation in the CARMIL2 gene: NM_001317026.3, c.1865 C > T; p.Ala622Val. Parents were both heterozygous carriers. Based on the clinical picture of the second and third patients, the mutation explains the disease, and thus both patients need urgent bone marrow transplantation. See figure S5.

The fourth patient was a 14-year-old male who had a history of eczema, recurrent mastoiditis, blepharitis, recurrent warts on hands and feet, and seborrhea since the age of 6 months, presented with recurrent pustular and acneiform rash on his face and scalp that started 4 months prior to admission (Figure S3). The patient sought medical care multiple times and was treated for infections and skin lesions with antibiotics. There was positive family history for immunodeficiency CARMIL-2 mutation. The patient was allergic to sulfonamide as he developed severe skin rash when treated with. His immunizations were up-to-age. He was started on prophylactic medications including azithromycin and itraconazole, in addition to selenium sulfide shampoo for seborrheic dermatitis. As the patient had a family history of CARMIL2 mutation and to rule out immunodeficiency, WES was ordered and confirmed SCID due to homozygote mutation C > T c. 1973 C > T p. Ala658Val Exon 21/38 missense in the CARMIL2 gene. Based on the clinical picture of the patient, the mutation explains the disease, and thus the patient needs bone marrow transplantation.

The fifth patient was a 14-year-old female whose parents are cousins, had a history of recurrent scalp abscesses associated with an itchy rash over flexural surfaces that started one year ago. On physical examination, there was a pustular rash on her hands, feet, and elbows (Figure S4); the rest of her examination was unremarkable. As an infant, the patient had a history of failure to thrive and experienced recurrent episodes of itchy skin rashes all over her body that used to disappear spontaneously. Her immunizations were up-to-age, and she had a positive family history of immunodeficiency (CARMIL-2 mutation). The patient sought medical advice and was treated for skin infections with antibiotics. Based on her clinical manifestations and the positive family history, the patient was suspected to have SCID. She was started on permethrin, ivermectin, and fexofenadine, and was referred to do WES testing to confirm the diagnosis which showed CARMIL2 gene mutation NM_001317026.3, c.1865 C > T; p.Ala622Val. Her laboratory findings are shown in (Table S2).

## Discussion

The presented case series provides a comprehensive overview of five individuals diagnosed with CARMIL2 mutations, explaining the diverse clinical manifestations associated with this rare genetic disorder. The uniqueness of the study lies in its documentation of the first reported case of recurrent visceral leishmaniasis attributed to a CARMIL2 mutation, emphasizing the importance of genetic investigations in understanding complex immunodeficiency disorders.

CARMIL2 is a regulator of actin capping protein which is a critical component of cell motility [9]. It is observed in the skin, lymphoid tissue, and gastrointestinal system [6]. T-cell lymphoblasts lacking CARMIL2 protein demonstrate inadequate distribution of actin along their leading edges and impaired microtubule networks. While these cells exhibit heightened speed in migration experiments, they show reduced capability for chemokine-directed movement. This underscores the crucial role of CARMIL2 protein in organizing the cytoskeleton of T-cells [8]. 

Beyond differences in observable characteristics, CARMIL2 protein deficiency is characterized by notable genetic heterogeneity. The identified variants associated with CARMIL2 mutation have distinct impacts on the protein, including nonsense, frameshift, missense, etc., and are distributed across the gene. CARMIL2 gene contains many genetic domains in which different mutations may occur. The most commonly involved domain is the leucine-rich repeat (LRR) domain, a conserved motif of 20–30 amino acids found in various species, known for its role in protein–protein interactions. In CARMIL2, the LRR is instrumental in directing the peptide to vimentin intermediate filaments. The repetitive motif forms a curved solenoid structure, stabilized by a leucine-rich hydrophobic core. Noteworthy is the observation that most reported autosomal recessive missense mutations in CARMIL2 gene specifically affect leucine residues within the LRR domain. Another frequently reported domain is the pleckstrin homology (PH) domain which is typically involved in membrane localization and contributes to anchoring the protein to filaments along with LRR [1]. 

Consistently, mutations are absent in the C-terminus of the gene, suggesting that mutations near the end of the coding sequence may preserve some level of protein function. This observation highlights the potential for missed diagnoses when the expected phenotype is not manifested. The complex and diverse clinical presentations of CARMIL2 deficiency, coupled with the lack of straightforward connections between genetic variations and observable traits, imply the involvement of additional factors such as environmental influences, genetic variations, or epigenetic modifications in shaping the clinical outcomes of this deficiency [10]. 

Our case series consisted of three male and two female patients, with consanguinity observed in two families. A review of previous literature on CARMIL2 mutations reveals that the clinical manifestations differ depending on the specific mutation [5], with recurrent pulmonary infections, dermatitis, skin abscesses, molluscum contagiosum, warts, and signs of EBV infection being the most common features [1]. 

In our case series, skin involvement was a prominent clinical feature, presenting as warts, chronic dermatitis, and skin abscesses in four patients with varying degrees of severity. Notably, two patients developed CMV-related infections, including CMV retinitis. Recurrent pulmonary infections were also observed in two cases. Additional infections including lymphadenitis, stomatitis, recurrent mastoiditis, blepharitis, sinusitis, esophagitis, and gastritis were also reported. These findings are congruent with most of the previous findings of reported CARMIL2-deficient patients. However, none of our patients experienced inflammatory bowel disease (IBD), or chronic diarrhea, which have often been reported in other patients with CARMIL2 mutations [5]. 

Of particular interest, our first patient presented with recurrent visceral leishmaniasis, right-sided vision loss due to CMV retinitis, herpes stomatitis, and suspected CMV colitis, which are distinct clinical presentations that were not reported in the previous literature. The processes by which different CARMIL2 variants propagate within the gene may result in varied immune phenotypes and remain not fully understood.

Four of the presented cases were found to have NM_001317026.3, c.1865 C > T; p.Ala622Val homozygous mutation, and one patient was found to have C > T c. 1973 C > T p. Ala658Val homozygous mutation. Interestingly, these two are considered novel mutations in CARMIL2 gene as they were not reported previously in literature. Table S1 provides a comparative overview of the genetic and clinical features observed in our patients alongside those reported in the literature. Table S2 show the clinical presentations and laboratory results of patients in this study.

An additional significant finding in our cohort is that the same rare homozygous CARMIL2 mutation (NM_001317026.3, c.1865 C > T; p.Ala622Val) was identified in four of the five patients. Two of these patients are siblings, while the other two belong to the same extended family as more distant relatives. This genetic grouping within single extended family strongly suggests the presence of a founder effect.

The founder effect is a well-known genetic phenomenon that describes how a mutation from a single common ancestor can enrich a genetically or geographically isolated group across generations. Even in the absence of close consanguinity, founder mutations can recur in several families within the same extended family, particularly in populations where marriage within extended families is culturally common. This mechanism may explain the recurrent CARMIL2 mutation among our patients [11, 12]. 

It is believed that immune modulation has helped stabilize the impaired immune function. Currently, there are no targeted therapies that directly address the immune pathway dysfunction caused by CARMIL2 mutation, making management of this primary immunodeficiency markedly limited. Patients usually need urgent allogeneic hematopoietic stem cell transplantation as this is considered one of the acceptable and curative treatments for CARMIL2 gene mutations. However, the lack of long-term outcome data prevents the routine use of this treatment [5]. 

In essence, CARMIL2 deficiency exhibits a diverse spectrum of clinical presentations, extending beyond known manifestations to include severe immunodeficiency and organ-specific autoimmune disorders. It is crucial to consider CARMIL2 as part of the diagnostic evaluation for individuals suspected of having recurrent respiratory, mucocutaneous, and atypical infections such as recurrent visceral leishmaniasis.

## Conclusion

Our study marks a significant contribution to the understanding of CARMIL2-related immunodeficiency, particularly in the Palestinian region. By reporting the first documented case of recurrent visceral leishmaniasis linked to CARMIL2 mutation, the research expands the global knowledge base of this rare disorder and its variable clinical presentations.

## Supplementary Information

Supplementary material 1.

## Data Availability

No datasets were generated or analysed during the current study.
