# Supplementary material for: Combined Immunodeficiency Associated with Two Novel CARMIL2 Mutations: A Case Series
Source: J Clin Immunol. 2025 Nov 10;45(1):156. doi: 10.1007/s10875-025-01956-1 (PMC12597840; doi:10.1007/s10875-025-01956-1)
Supplement: Supplementary file 1 — Supplementary material 1. [file 10875_2025_1956_MOESM1_ESM.docx]

Table S1: Clinical Presentations of Patients with CARMIL-2 Mutation in Different Studies‎

| **Table S1** | Sorte et al.^7^ | Schober et al.^8^ | Wang et al.^11^ | Kurolap et al.^12^ | Maccari et al. ^13^ | Alazami et al.^1^ | This study |
| --- | --- | --- | --- | --- | --- | --- | --- |
| Total patients | 4 | 4 | 6 | 1 | 1 | 7 | 5 |
| Age | 18-52 | 4-14 | 2-27 | 0/1 | 0/1 | 4-34 | 13-37 |
| Sex | 2M/2F | 2M/2F | 2M/4F | 0/1 | 0/1 | 4M/3F | 3M/2F |
| Variants | CARMIL-2(c.1916T>A:p.Leu639His) | CARMIL-2(c. 489insG:p.E163fsX4)  CARMIL2(c.871 +1G>T:p.D260fsX70) | - | CARMIL-2(c.1590C>A:p.Asn530Lys) | CARMIL-2 (c.1071+1G>T)  PLEC1(c.7468C>T:p.Gln2490*) | - | - NM_001317026.3, c.1865C>T; p.Ala622Val genomic position (Hg38): Chr. 16, 67683762 - C>T c. 1973C>T p. Ala658Val Exon 21/38 |
| Consanguinity | 0/4 | 4/4 | 5/6 | 0/1 | 1/1 | 7/7 | 4/5 |
| Recurrent chest infections | 3/4 | 4/4 | 5/6 | 0/1 | 1/1 | 5/7 | 2/5 |
| Dermatitis | 4/4 | 2/4 | 5/6 | 0/1 | 1/1 | 6/7 | 4/5 |
| Warts | 4/4 | 2/4 | 0/6 | 0/1 | 0/1 | 2/7 | 3/5 |
| Skin abscess | 3/4 | 1/4 | 2/6 | 0/1 | 0/1 | 7/7 | 2/5 |
| Mucocutaneous Candidiasis | 0/4 | 0/4 | 3/6 | 0/1 | 0/1 | 2/7 | 1/5 |
| Esophageal disease | 0/4 | 0/4 | 1/6 | 0/1 | 0/1 | 5/7 | 1/5 |
| Cytomegalovirus related infections | 0/4 | 0/4 | 0/6 | 0/1 | 0/1 | 0/7 | 2/5 |
| Epstein-Barr virus-related smooth muscle tumor | 0/4 | 4/4 | 0/6 | 0/1 | 0/1 | 0/7 | 0/5 |
| Mollascum contagiusm | 2/4 | 0/4 | 1/6 | 0/1 | 0/1 | 0/7 | 0/5 |
| Visceral leishmaniasis | 0/4 | 0/4 | 0/6 | 0/1 | 0/1 | 0/7 | 1/5 |
| Diarrhea/ IBD | 0/4 | 0/4 | 0/6 | 1/1 | 1/1 | 0/7 | 0/5 |
| Lymphadenitis/ generalized lymphadenopathy | 0/4 | 0/4 | 0/6 | 0/1 | 1/1 | 0/7 | 1/5 |
| Failure to thrive | 0/4 | 0/4 | 0/6 | 1/1 | 1/1 | 0/7 | 1/5 |
| Mortality | 0/4 | 3/4 | 1/6 | 0/1 | 0/1 | 0/7 | 1/5 |

Table S2: Clinical Presentations and Laboratory Results of Patients with CARMIL-2 Mutation in this Study.

| **Table S2** | First Patient | Second Patient | Third Patient | Fourth Patient | Fifth Patient |
| --- | --- | --- | --- | --- | --- |
| Age (years) | 37 | 16 | 14 | 14 | 14 |
| Sex | Male | Female | Male | Male | Female |
| Age of diagnosis | 18 | 15 | 13 | 14 | 13 |
| Chest infection | no | yes | yes | no | No |
| Warts | no | yes | yes | yes | No |
| Dermatitis | no | yes | yes | yes | Yes |
| Candida esophagitis, dysphagia, and odynophagia | no | no | yes | no | No |
| Other Clinical Presentations | Recurrent visceral leishmaniasis, hepatosplenomegaly, sublingual and submandibular lymphadenopathy, herpes stomatitis, CMV retinitis and suspected CMV colitis. | - | Upper limit of normal liver size, sinusitis, H. pylori infection, active and chronic gastritis, EBV and CMV infections. | Recurrent mastoiditis, blepharitis, and seborrhea. | Recurrent scalp abscess |
| Labs | | | | | |
| Hemoglobin | low | - | low | normal | - |
| White blood cell count (cell/uL) | low | - | high | normal | Normal |
| Platelets | low | - | high | 247 | - |
| Lymphocyte count (cell/uL) | 782 | 4373 | - | 4500 | 5391 |
| T-cells (cell/uL) | 328 | 3129 | - | - | 3990 |
| B-cells (cell/uL) | 406 | 1006 | - | - | 1240 |
| NK cells (%) | 11% | 7% | - | - | - |
| CD4 (cell/uL) | 117 | 1880 | - | - | 2642 |
| CD8 (cell/uL) | 195 | 1093 | - | - | 1024 |
| HLA-DR | Normal | Normal | - | - | Normal |
| IgG (mg/dl) | 401 | 1070 | 1191 | 682 | 905 |
| IgA (mg/dl) | 90.7 | 157 | 159 | 164 | 161 |
| IgM (mg/dl) | 58.2 | 263 | 216 | - | 126 |
| IgE (mg/dl) | <4.54 | - | 20.44 | - | - |
| TREC levels | 32 | - | - | - | 1771 |
| CGD assay | Normal | Normal | - | - | - |
| EBV IgG | - | - | Reactive 335.9 | - | - |
| EBV IgM | - | - | Nonreactive 0.367 | - | - |
| CMV IgG | - | - | Reactive 39.31 | - | - |
| CMV IgM | - | - | Borderline -0.733 | - | - |

**Figures**


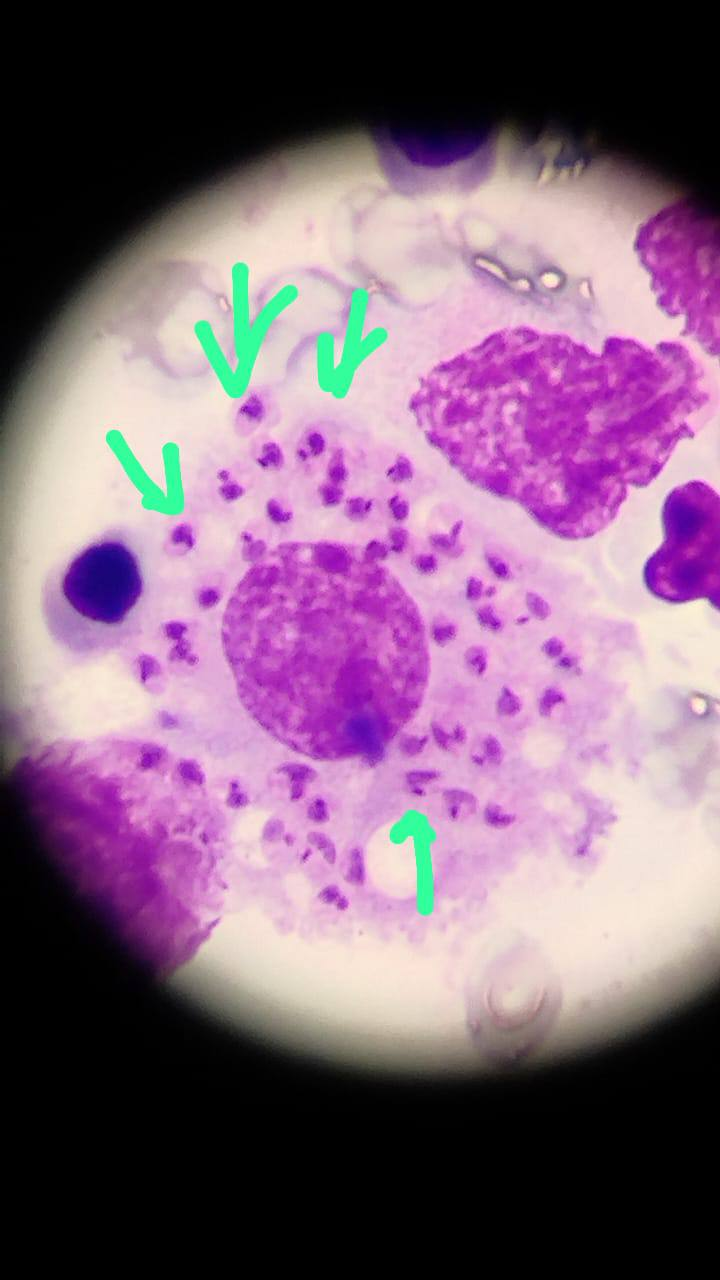


**Figure S1: Bone marrow biopsy showing macrophages with amastigotes/Denovan bodies (green arrows).**


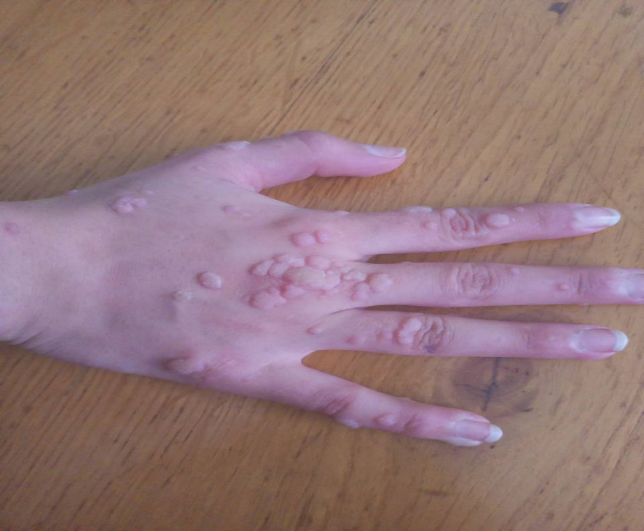


**Figure S2: Multiple warts involving the patient's hand.‎**


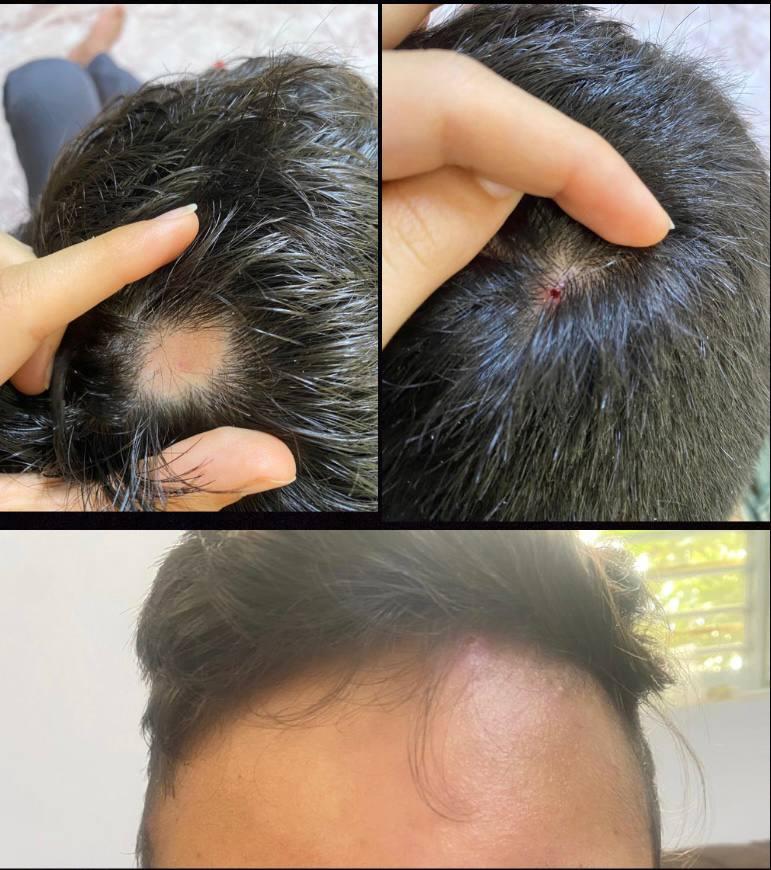


Figure S3: Skin lesions involving the patient's scalp and face.‎


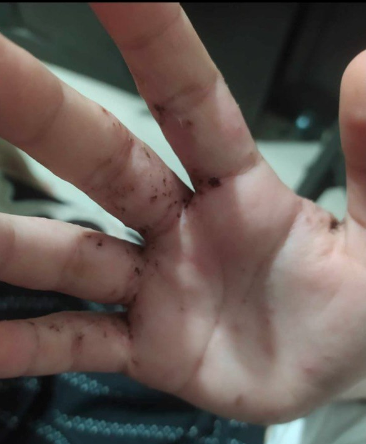


**Figure S4: pustular rash on fingers and hand.**


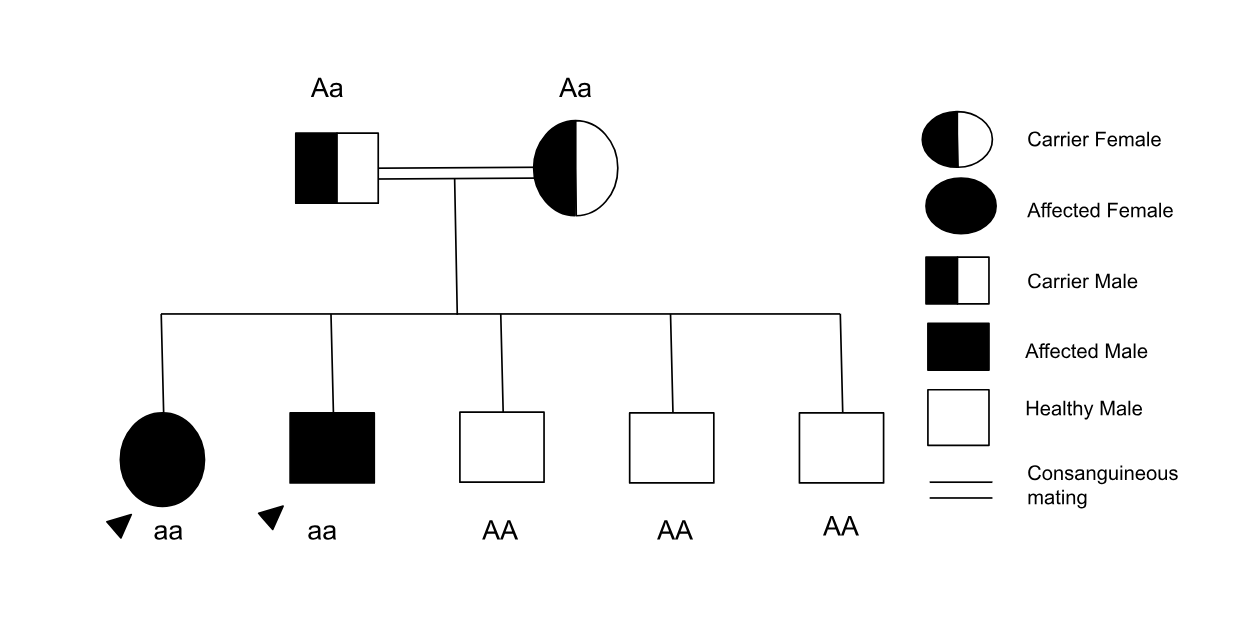


Figure S5: Pedigree illustrating the inheritance of the CARMIL 2 gene mutation in the family of the second and third patients, consistent with an autosomal recessive mode of inheritance.
